# Dynamic postural control and associated attentional demands in contemporary dancers versus non-dancers

**DOI:** 10.1371/journal.pone.0173795

**Published:** 2017-03-21

**Authors:** Geneviève Sirois-Leclerc, Anthony Remaud, Martin Bilodeau

**Affiliations:** 1 School of Rehabilitation Sciences, Faculty of Health Sciences, University of Ottawa, Ottawa, Canada; 2 Interdisciplinary School of Health Sciences, Faculty of Health Sciences, University of Ottawa, Ottawa, Canada; 3 Bruyère Research Institute, Ottawa, Canada; 4 School of Human Kinetics, Faculty of Health Sciences, University of Ottawa, Ottawa, Canada; Universita degli Studi di Roma 'Foro Italico', ITALY

## Abstract

Postural control is not a fully automatic process, but requires a certain level of attention, particularly as the difficulty of the postural task increases. This study aimed at testing whether experienced contemporary dancers, because of their specialized training involving the control of posture/balance, would present with a dual-task performance suggesting lesser attentional demands associated with dynamic postural control compared with non-dancers. Twenty dancers and 16 non-dancers performed a dynamic postural tracking task in both antero-posterior and side-to-side directions, while standing on a force platform. The postural task was performed, in turn, 1) as a stand-alone task, and concurrently with both 2) a simple reaction time task and 3) a choice reaction time task. Postural control performance was estimated through variables calculated from centre of pressure movements. Although no overall group difference was found in reaction time values, we found a better ability to control the side to side movements of the centre of pressure during the tracking task in dancers compared with non-dancers, which was dependent on the secondary task. This suggests that such increased ability is influenced by available attentional resources.

## Introduction

Dynamic postural control can be defined as the ability to maintain the center of mass within the base of support while the body is subjected to internal or external perturbations that are anticipated or not [1]. The constant integration of afferences from peripheral sensory systems (vision, vestibular and prioprioceptive) by the central nervous system is necessary to achieve optimal postural control [2, 3]. Furthermore, it is now widely accepted that the regulation of posture is a process that is not completely automatic, but which requires a certain amount of attentional resources [3, 4–7].

Studies using the dual-task paradigm to investigate changes in attentional demands associated with selected postural/balance activities have led to the documentation of an increase in attentional demands with increasing task difficulty [4, 5, 8] or in selected groups of individuals with altered abilities (e.g., older adults [9, 10]). For example, several studies using a reaction time task as a secondary cognitive task performed concurrently with a primary postural task, have shown increased attentional demands (reflected in an increase in reaction time) with more demanding postural tasks, such as standing versus sitting [4], single support versus double support phases in walking [5] and even standing quietly on one leg versus with feet together or in semi-tandem [8]. Furthermore, it has been suggested that the difficulty of the secondary (cognitive) task can also have an influence on posture control, with more complex tasks being potentially more detrimental to posture than easier ones [7].

Conversely, attentional demands associated with postural control could be decreased if an individual becomes more proficient at performing a given task, such as in elite athletes (e.g., gymnasts [6]). According to Boisgontier et al. [3], this has not yet been the focus of systematic investigation. Although it has been reported that practicing a given sport can have an influence on postural control [3, 11], it is not clear whether attentional demands associated with postural tasks would be modulated in individuals who have been trained in executing complex postural tasks. Vuillerme & Nougier [6] provided preliminary evidence of such possible modulation by showing that gymnasts showed a lesser increase in attentional demands (increase in simple reaction time) with increasing postural task difficulty compared with non-gymnasts.

Individuals with a documented expertise in dancing (e.g., ballet or other) have been shown to present with different postural control capabilities compared with individuals without dancing expertise [12–15], although specific differences vary according to the task performed (static versus dynamic) or the condition tested (e.g., eyes opened versus closed). The hypothesis we wanted to test in the present study was whether experienced contemporary dancers, because of their specialized training involving the control of posture/balance, would present a dual-task performance suggesting lesser attentional demands associated with dynamic postural control compared with non-dancers.

## Methodology

### Participants

Twenty experienced dancers (17 women; age: 23 ± 3 years; height: 169 ± 6 cm; weight: 62.8 ±8.9 kg) and 16 non-dancers (13 women; age: 22 ± 2 years; height: 165 ± 10; weight: 65.2 ± 12.6 kg), all healthy adults, participated in the study. None of the participants reported a history of vestibular or lower limb orthopaedic injuries in the six months prior to data collection. For the dancer group, all participants were current students or recent graduates of the Contemporary Dance Diploma Program at The School of Dance in Ottawa, a registered private career college. The program is a 3-year, 35 hours per week training curriculum which includes Modern and Contemporary technique classes. Dancers had been practicing the contemporary style for an average of 6.0 ± 3.9 years, and multiple other dance styles intensively for 7.9 ± 3.8 years. For the non-dancer group, participants were selected by convenience and none had received formal dance training. Participants (non-dancers) who did participate in dancing activities had done so recreationally for 1–5 years during childhood or adolescence. All non-dancers were physically active, participating in various sports (running/walking (n = 10), soccer (n = 6), cycling (n = 5), hockey/skating (n = 5), swimming (n = 4), cardio (n = 3), volleyball (n = 2), other (n = 5)) for an average of 4.7 ± 3.1 hours/week. Before the testing session, participants were informed of the procedures and signed a written consent form. The experimental protocol of this study was approved by the University of Ottawa and Bruyère Continuing Care Research Ethics Boards.

### Materials and procedures

The experimental protocol comprised the following tasks, in order:

#### 1) Familiarization with the cognitive tasks

Two cognitive tasks were used in the study: 1) a simple reaction time (SRT) task required participants to verbally respond «TOP» as fast as possible following randomly timed auditory stimuli (50 ms duration at 3000 Hz); and 2) a choice reaction time (CRT) task required participants to verbally respond «TY» following a high-pitched auditory stimulus (50 ms duration at 3000 Hz) or «TOE» following a low-pitched auditory stimulus (50 ms duration at 250 Hz). For the CRT task, the presentation of high- versus low-pitched stimuli in a given trial was randomized. Participants were introduced to these tasks in sitting, and asked to focus on a point on the wall in front of them. The verbal responses and auditory stimuli were recorded with a hands-free microphone secured to the participant’s clothing at the level of the sternum (Model: BH-212; Nokia Corporation, Newmarket, ON, Canada) and WavePad Sound Editor software (version 4.27, NCH Software Pty Ltd, Canberra, Australia). Two familiarization trials lasting 30 s (6 auditory stimuli/trial) were performed for each of the SRT and CRT tasks.

#### 2) Measurement of baseline reaction times in static standing

Baseline measures of SRT and CRT were taken while the participant was standing still on an AcuGait force platform (AMTI, Watertown, MA, USA), bare feet together, hands on the hips and eyes looking forward. Four 30-s baseline trials (2 SRT trials and 2 CRT trials; 6 stimuli/trial) were recorded.

#### 3) Familiarization with the dynamic postural task and dual-task conditions

Center of pressure (COP) displacements during dynamic postural control tasks were recorded using the same force platform as above. Each participant’s base of support was first determined using the *Balance Trainer* software (version 1.05.02, AMTI, Watertown, MA, USA). Participants were asked to stand barefoot on the force platform (same position as above), and to lean as far as possible anteriorly and posteriorly, and side-to-side without losing their balance and maintaining contact between the sole of the foot and the platform at all times. Participants were instructed to move about their ankles and keep knees and hips straight. The greatest excursions in both the antero-posterior (AP) and side-to-side (medio-lateral: ML) directions were used to determine the limits of the base of support. Then, on a screen in front of them, the software allowed participants to see a representation of their base of support, their COP, and a target (circle). This target had a diameter of 2.5% of the base of support for a given participant, and moved at a speed of 1.9 cm/s, either in the AP or ML directions, within limits set at 80% of the base of support. The dynamic postural task consisted in participants having to follow the target’s movements in the AP or ML direction, keeping their COP as close as possible to the center of the target, for a pre-determined number of movement cycles which allowed for similar trial duration in both directions (4 complete cycles for AP and 7 complete cycles for ML, because of a smaller base of support length for the latter direction). During familiarization, each possible condition combination was practiced once in each direction: postural task alone, postural task with SRT task, and postural task with CRT task. Before the start of each trial, participants were asked to position their COP in the center of the target, and were informed of which direction the target would be moving (AP or ML).

#### 4) Measurements of postural and cognitive parameters in dynamic postural tasks (under single and dual-task conditions)

A total of 24 randomized trials were administered during the testing protocol: 4 trials of each condition, in both directions. Therefore, each participant completed 8 trials of postural task without reaction times (4 x AP and 4 x ML), 8 trials of postural task with SRT (4 x AP-SRT and 4 x ML-SRT), and 8 trials of postural task with CRT (4 x AP-CRT and 4 x ML-CRT). As was done for familiarization trials, participants adopted the standardized position, were informed of the upcoming direction of target movement, and were reminded of the condition (no reaction time, SRT or CRT). Participants were asked to prioritize the postural task (primary) over the cognitive task (secondary).

### Data processing and statistical analysis

#### Reaction time

Reaction time was defined as the time, in s, between the beginning of a given auditory stimulus and the beginning of a participant’s verbal response. They were measured semi-automatically using a Matlab script (Mathworks, Natick, MA, USA), where the beginning of stimuli and verbal responses were defined and identified as an increase of the auditory signal intensity above the base signal plus 5 standard deviations [16]. Reaction times identified as outliers (Grubb’s test) as well as wrong answers to auditory stimuli were excluded from statistical analyses (e.g. responding TOE instead of TY); in dancers, 68 of 3813 and in non-dancers, 59 of 3128 reaction times were eliminated, representing < 2% of all reaction times in each group. In order to best depict the increase in attentional demands associated with the dynamic postural task, SRT and CRT obtained during this task were expressed as a percent increase or decrease of the value obtained during the baseline quiet standing condition, which was then used in the statistical analyses.

#### Stabilometry

Each participant’s 24 postural trials were individually analyzed and characterized by calculating: 1) the average distance between the COP and the target’s center (proximity to the target, in cm—reflecting how well participants were able to perform the task) and 2) the average velocity of the COP (sum of all COP displacements/trial duration; in cm/s—reflecting the extent of corrective actions needed to follow the target and maintain balance).

Independent t-tests were used to assess differences between dancers and non-dancers in anthropometric measures and baseline reaction times. Three-way analyses of variance (ANOVAs) for mixed designs were used to test the effects of 1) group (dancers/non-dancers), 2) task (for stabilometric variables: single task (no reaction time)/dual-task with SRT/dual-task with CRT; for change in reaction time from baseline: dual-task with SRT/dual-task with CRT) and 3) target direction (AP/ML) on stabilometric variables and percent change in reaction time from baseline. When the sphericity assumption in repeated measures ANOVAs was violated (Mauchly’s test), a Geisser/Greenhouse correction was used. If relevant, *post hoc* tests were performed by means of the Newman-Keuls procedures. Statistical significance was set at *p*< 0.05. All results presented in the text are mean ± standard deviation.

## Results

Independent t-tests confirmed that dancers and non-dancers were not different in terms of sex, age, height, weight and base of support (p>0.05).

### Reaction time

**Fig 1:**
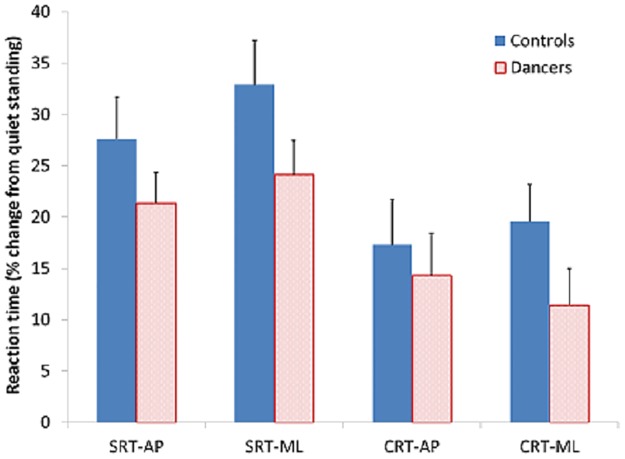
Reaction time. Percent change in reaction time from the quiet standing baseline condition for both the antero-posterior (AP) and side-to-side (ML) dynamic tracking tasks. Values for both the simple reaction time (SRT) and choice reaction time (CRT) secondary tasks are contrasted between dancers and non-dancers. Bars represent group mean plus standard error.

Table 1 shows the absolute reaction times and corresponding percent change from baseline values for both dancers and non-dancers. There was no difference between dancers and non-dancers in SRT or CRT for the baseline quiet standing condition (SRT: t_(34)_ = 0.67, p = 0.51; CRT: t_(34)_ = 0.63, p = 0.53). Baseline SRT values (299 ± 58 ms) were also significantly shorter than CRT values (388 ± 105 ms; paired-t_(35)_ = -9.20, p<0.001), reflecting the greater attentional demands associated with the CRT task. Fig 1 shows that the percent increase in both SRT and CRT from the quiet standing condition to the dynamic tasks, suggesting an increase in attentional demands for the latter, was somewhat more pronounced for the non-dancers, particularly for the ML direction. However, these tendencies did not reach statistical significance (main group effect (F_(1,34)_ = 1.94, p = 0.17; group × direction interaction, F_(1,34)_ = 2.94, p = 0.095). Only the effect of task (SRT/CRT) was significant (F_(1,34)_ = 25.81, p<0.001), with the percent increase in reaction time from baseline being more pronounced for SRT than CRT.

**Table 1:** Mean reaction time (± standard deviation) for dancers and non-dancers during quiet standing baseline trials and across the different dynamic postural conditions.

	SRT (in ms)					CRT (in ms)				
	*Baseline*	*AP*	*Δ (%)*	*ML*	*Δ (%)*	*Baseline*	*AP*	*Δ (%)*	*ML*	*Δ (%)*
Dancers (n = 20)	293 ± 37	354 ± 49	21 ± 13	362 ± 50	24 ± 15	378 ± 57	429 ± 76	14 ± 18	418 ± 70	11 ± 16
Non-dancers (n = 16)	307 ± 78	392 ± 116	28 ± 16	410 ± 130	33 ± 17	401 ± 146	467 ± 171	17 ± 18	476 ± 168	20 ± 14

AP: antero-posterior; ML: side-to-side (medio-lateral); SRT: simple reaction time; CRT: choice reaction time.

### COP proximity

**Fig 2:**
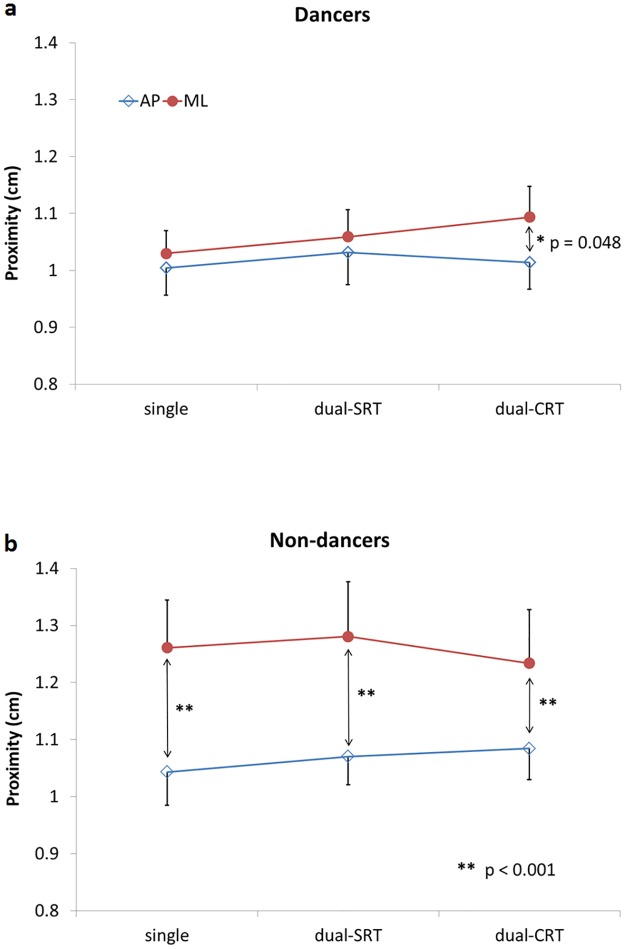
COP proximity. Centre of pressure (COP) proximity (cm) to the target plotted against the dual-task condition (single task, SRT dual-task, CRT dual-task) for both the antero-posterior (AP) and side-to-side (ML) tasks. Results from dancers (top panel) and non-dancers (bottom panel) are contrasted. Data points represent group mean plus or minus standard error.

Overall, participants were able to keep their COP representation within about 1 cm of the center of the moving target. The ANOVA for the proximity variable revealed a significant effect of direction (F_(1,34)_ = 10.55, p = 0.003) and significant interactions between group and direction (F_(1,34)_ = 4.17, p = 0.049) and group, direction and task (F_(2,68)_ = 3.66, p = 0.031). As shown in Fig 2, posthoc analyses revealed that proximity was only different between AP and ML directions in dancers for the CRT task (7.8% further from the target in ML versus AP; p = 0.048). In contrast, proximity was significantly different between AP and ML for all tasks in non-dancers (COP further from target in ML versus AP by 20.9% for the single task (p<0.001), 19.7% for SRT task (p<0.001) and 13.8% for the CRT task (p<0.001)).

### COP velocity

**Fig 3:**
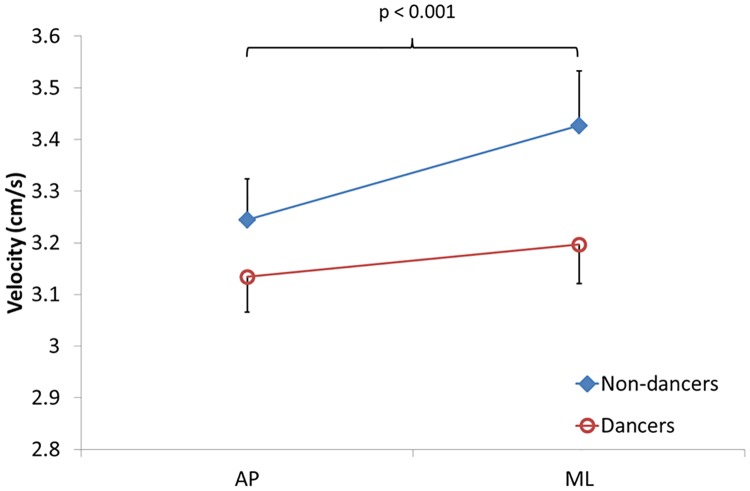
COP velocity. Centre of pressure (COP) velocity (cm/s) plotted for both the antero-posterior (AP) and side-to-side (ML) tasks. Results from dancers and non-dancers are contrasted. Data points represent group mean (with values from single task, SRT dual-task and CRT dual-task polled together) plus or minus standard error.

For COP velocity, a main effect of direction (greater velocity in ML versus AP; F_(1,34)_ = 23.09, p<0.001) and a group × direction interaction (F_(1, 34)_ = 5,53; p<0.025) were found significant. From Fig 3, it can be seen that COP velocity was somewhat higher in non-dancers than in dancers; however, this difference was not significant (no main effect of group). Posthoc tests for the interaction indicated that COP velocity was not different between AP and ML for dancers (p = 0.092), but was 5.6% greater in ML than AP in non-dancers (p<0.001). A direction × task interaction was also found (F_(2, 68)_ = 7,93; p<0.001). Posthoc tests showed that in AP, velocity was significantly higher for SRT compared with the single task (p = 0.006), whereas for ML, velocity was decreased during CRT compared with the single task (p = 0.008).

## Discussion

This study aimed at documenting potential differences in dual-task performance during dynamic postural control between experienced contemporary dancers and non-dancers. The main findings of this study were: a better ability to control ML as well as AP COP movements in dancers compared with non-dancers, which was dependent on the secondary task; and, a tendency for a less pronounced increase in RT during the dynamic task compared with the static baseline in dancers compared with non-dancers.

The significant interactions between the group and direction factors for both the proximity and COP velocity variables suggest that the control of COP movements was different for dancers compared with non-dancers. Proximity values were generally greater, i.e. the COP representation was further from the target, for tasks involving ML target movements compared to AP movements in non-dancers, reflecting the fact that performance for the tracking task was worse for the ML direction in this group (Fig 2). COP velocity also increased from AP trials to ML trials in those participants, suggesting they needed more postural corrections to perform the dynamic postural task in the latter direction (Fig 3). In comparison, the performance of dancers was similar for ML and AP target movement tasks, except under the CRT dual-task condition, where proximity values were somewhat greater for ML target movements. Thus, our data indicates that dancers and non-dancers presented a similar ability to control their COP in a dynamic tracking task when the target was moving in the AP direction. Interestingly, the performance of non-dancers deteriorated when the target was moving from side to side (ML), regardless of the dual-task condition. In contrast, dancers controlled their COP with the target moving in ML as well as when moving in AP for the postural task performed without a RT task and the SRT dual-task. It is only with the addition of the more (attentionally) demanding CRT task that their performance worsened for ML target movements compared with AP movements. This dual-task interference may reflect an overload of the attentional resources, according to the limited capacity theory of attention [17, 18]. Alternatively, according to the "bottleneck" theory [19, 20], this may also suggest that both the postural and RT tasks could not be processed at the same time and that one task (the CRT task) had to be delayed. The fact that greater attentional demands were needed for the CRT task is indicated by the significantly longer reaction times obtained in this condition compared with the SRT condition (Table 1). The improved performance for ML target movements in dancers during the conditions requiring less attention reflects their greater postural control abilities. In contrast, the fact that the performance of non-dancers was similarly poorer in the ML target direction tasks compared with the AP tasks, irrespective of the secondary task, likely reflects the generally higher difficulty level of the task for this group, irrespective of the secondary task.

The greater ability to control COP movements in the ML as well as the AP direction in dancers compared with non-dancers is likely a consequence of their specialized training. For example, Bruyneel, Mesure, Paré, & Bertrand [21] report decreased ground reaction force oscillations and increased variability in the ML but not the AP plane, and a lesser number of falls (loss of balance) following a leg lift in older, more experienced ballet dancers compared with younger, less experienced ones. This may be associated with the fact that “training the dynamic equilibrium of (ballet) dancers is mainly done in the upright position …, and on a frontal plane…” [21], which we think would particularly increase postural control abilities in the ML direction. Rein, Fabian, Zwipp, Rammelt, & Weindel [22] also found a different postural control strategy between experienced dancers and less experienced dancers or non-dancers. Experienced dancers were found to balance on an unstable surface mostly on the antero-lateral and less on the postero-medial aspect of their foot compared with amateur dancers and controls. Interestingly, the enhanced control in the ML direction may be specific to the type of dance expertise. In contrast to the present results, Kuczynski, Szymanska, & Biec [23] compared postural sway during quiet standing between competitive ballroom dancers and non-dancers and found similar sway characteristics for the ML plane, but greater sway variability in non-dancers for the AP plane under a dual task condition. Ballroom dancing would typically involve variable-speed displacements forward, backward and side to side, as well as turns.

The abov[e differences between dancers and non-dancers were expected and complement previous work showing better postural control in individuals with extensive training in activities requiring balance, such as gymnasts [6] and dancers [12–15]. In addition to differences in postural control variables, we expected to find differences in the secondary task, potentially indicating lesser attentional demands required to perform the dynamic tracking task in dancers compared with non-dancers. Although the reaction time results showed a trend in line with this hypothesis, with a somewhat lesser increase in reaction time from the quiet standing baseline to the dynamic tasks in dancers compared with non-dancers (with the difference being more pronounced for the ML dual-task), this trend did not reach statistical significance. The increase in reaction time from quiet standing to the dynamic task was relatively important (10–30% or 50–100 ms), indicating that the latter task required a significant amount of attention. In contrast, we have previously reported an increase in SRT of about 30 ms from the same quiet standing task to a challenging task requiring standing on one foot with eyes closed [8]. The SRT and CRT increases in the present study are not surprising as the dynamic postural task involved tracking, which required the participant to continuously focus on both the target and the COP representation. This, and the fact that reaction time tasks may not require a great amount of attentional resources, may have prevented more obvious group differences from being evidenced in the secondary task.

Overall, the results of the present study showed that experienced contemporary dancers can control the movement of their COP during a dynamic tracking task as well in the ML than the AP direction, unlike non-dancers whose performance decreases for the ML direction. This increased ability in dancers, however, was present when they performed the tracking task alone or together with a SRT task, but was lost when the tracking task was performed in conjunction with a CRT secondary task. This suggests that such increased ability is dependent on available attentional resources.

These findings suggest that a specialized training involving the control of posture/balance, such as dancing, results in a greater ability to perform a postural task concurrently with a cognitive activity. Such an increased dual-tasking ability with expertise/training has also been observed in experienced baseball batters [24], soccer players [25] and shooters [26]. This may have some implications for specific populations such as older adults for whom activities such as dancing may be recommended in order to preserve their balance in multiple contexts. Recently, Hamacher et al. [27] reported improvements in dual-task performance (gait and backwards counting) after a dancing program, compared to a traditional exercise program. Thus, future studies should focus on the potential benefit of activities such as a dancing program as a mean of improving balance and dual-task ability in populations with impaired balance and/or attentional deficit such as the elderly.

## Supporting information

S1 Data (XLSX)

## References

[1] Winter DA, Patla AE, Frank JS. Assessment of balance control in humans. Med Prog Technol. 1990; 16: 31–51. 2138696

[2] Teasdale N, Bard C, Larue J, Fleury M. On the cognitive penetrability of posture control. Exp Aging Res. 1993; 19(1): 1–13. 10.1080/03610739308253919 8444263

[3] Boisgontier M, Mignardot JB, Nougier V, Olivier I, Palluel E. Le coût attentionnel associé aux fonctions exécutives impliquées dans le contrôle postural. Mov Sport Sci. 2011: 3: 53–64.

[4] Lajoie Y, Teasdale N, Bard C, Fleury M. Attentional demands for static and dynamic equilibrium. Exp Brain Res. 1993; 97: 139–144. 8131825 10.1007/BF00228824

[5] Roerdink M, Hlavackova P, Vuillerme N. Center-of-pressure regularity as a marker for attentional investment in postural control: A comparison between sitting and standing postures. Hum Mov Sci. 2011; 30: 203–212. 10.1016/j.humov.2010.04.005 20542347

[6] Vuillerme N, Nougier V. Attentional demand for regulating postural sway: the effect of expertise in gymnastics. Brain Res Bull. 2004; 63(2): 161–165. 10.1016/j.brainresbull.2004.02.006 15130706

[7] Woollacott M Shumway-Cook A. Attention and the control of posture and gait: a review of an emerging area of research. Gait Posture. 2002; 16: 1–14. 12127181 10.1016/s0966-6362(01)00156-4

[8] Remaud A, Boyas S, Caron GA, Bilodeau M. Attentional demands associated with postural control depend on task difficulty and visual condition. J Mot Behav. 2012; 44(5): 329–340. 10.1080/00222895.2012.708680 22934664

[9] Brown LA, Shumway-Cook A, Woollacott MH. Attentional demands and postural recovery: the effects of aging. J Gerontol: Med Sci. 1999; 54A (4): M165–M171. 10.1093/gerona/54.4.m165 10219006

[10] Lajoie Y, Teasdale N, Bard C, Fleury M. Upright standing and gait: are there changes in attentional requirements related to normal aging? Exp Aging Res. 1996; 22: 185–198. 10.1080/03610739608254006 8735152

[11] Ericsson KA, Lehmann AC. Expert and exceptional performance: Evidence of maximal adaptation to task constraints. Annu Rev Psychol. 1996; 47 (1): 273–305. 15012483 10.1146/annurev.psych.47.1.273

[12] Hugel F, Cadopi M, Kohler F, Perrin P. Postural control of ballet dancers: a specific use of visual input for artistic purposes. Int J Sports Med. 1999; 20: 86–92. 10.1055/s-2007-971098 10190767

[13] Perrin P, Deviterne D, Hugel F, Perrot C. Judo, better than dance, develops sensorimotor adaptabilities involved in balance control. Gait Posture. 2002; 15: 187–194. 11869913 10.1016/s0966-6362(01)00149-7

[14] Schmit JM, Regis DI, Riley MA. Dynamic patterns of postural sway in ballet dancers and track athletes. Exp Brain Res. 2005; 163: 370–378. 10.1007/s00221-004-2185-6 15655686

[15] Simmons RW. Sensory organization determinants of postural stability in trained ballet dancers. Int J Neurosci. 2005; 115: 87–97. 15768854 10.1080/00207450490512678

[16] Remaud A, Boyas S, Lajoie Y, Bilodeau M. Attentional focus influences postural control and reaction time performances only during challenging dual-task conditions in healthy young adults. Exp Brain Res. 2013; 231(2): 219–229. 10.1007/s00221-013-3684-0 23995564

[17] Kahneman D. Attention and effort. Englewood Cliffs: Prentice-Hall; 1973.

[18] Wickens CD. Attention and skilled performance In: Holding DH, editor. Human skills. New York: John Wiley & Sons; 1989 pp. 71–105.

[19] Broadbent DE. Perception and communication. London: Pergamon Press; 1958.

[20] Pashler H. Dual-task interference in simple tasks: data and theory. Psychol Bull. 1994; 116: 220–244. 7972591 10.1037/0033-2909.116.2.220

[21] Bruyneel AV, Mesure S, Paré JC, Bertrand M. Organization of postural equilibrium in several planes in ballet dancers. Neurosci Lett. 2010;, 485: 228–232. 10.1016/j.neulet.2010.09.017 20849927

[22] Rein S, Fabian T, Zwipp H, Rammelt S, Weindel S. Postural control and functional ankle stability in professional and amateur dancers. Clin Neurophysiol. 2011; 122: 1602–1610. 10.1016/j.clinph.2011.01.004 21435946

[23] Kuczynski M, Szymanska M, Biec E. Dual-task effect on postural control in high-level competitive dancers. J Sports Sci. 2011; 29 (5): 539–545. 10.1080/02640414.2010.544046 21294035

[24] Gray R. Attending to the execution of a complex sensorimotor skill: expertise differences, chocking, and slumps. J Exp Psychol. 2004; 10: 42–54. 10.1037/1076-898X.10.1.42 15053701

[25] Smith MD, Chamberlin CG. Effect of adding cognitively demanding tasks on soccer skill performance. Percept Mot Skills. 1992; 75: 955–961. 10.2466/pms.1992.75.3.955 1454502

[26] Negahban H, Aryan N, Mazaheri M, Norasteh AA, Sanjari MA. Effect of expertise in shooting and Taekwondo on bipedal and unipedal postural control isolated or concurrent with a reaction-time task. Gait Posture. 2013; 38: 226–230. 10.1016/j.gaitpost.2012.11.016 23245642

[27] Hamacher D, Hamacher D, Rehfeld K, Hökelmann A, Schega L. The effect of a six-month dancing program on motor-cognitive dual-task performance in older adults. J Aging Phys Act. 2015; 23: 647–652. 10.1123/japa.2014-0067 25642826

